# Acute Exposure to Normobaric Hypoxia Impairs Balance Performance in Sub-elite but Not Elite Basketball Players

**DOI:** 10.3389/fphys.2021.748153

**Published:** 2021-10-27

**Authors:** Haris Pojskić, Helen G. Hanstock, Tsz-Hin Tang, Lara Rodríguez-Zamora

**Affiliations:** ^1^Department of Sports Science, Faculty of Social Sciences, Linnaeus University, Kalmar, Sweden; ^2^Department of Health Sciences, Faculty of Human Sciences, Mid Sweden University, Östersund, Sweden; ^3^Swedish Winter Sports Research Centre, Mid Sweden University, Östersund, Sweden; ^4^Division of Sport Science, School of Health Sciences, Orebro University, Orebro, Sweden

**Keywords:** postural control, high altitude training, oxygen saturation, team sports, single-leg balance test

## Abstract

Although high and simulated altitude training has become an increasingly popular training method, no study has investigated the influence of acute hypoxic exposure on balance in team-sport athletes. Therefore, the purpose of this study was to investigate whether acute exposure to normobaric hypoxia is detrimental to balance performance in highly-trained basketball players. Nine elite and nine sub-elite male basketball players participated in a randomized, single-blinded, cross-over study. Subjects performed repeated trials of a single-leg balance test (SLBT) in an altitude chamber in normoxia (NOR; approximately sea level) with FiO_2_ 20.9% and PiO_2_ ranging from 146.7 to 150.4 mmHg and in normobaric hypoxia (HYP; ~3,800 m above sea level) with FiO_2_ 13.0% and PiO_2_ ranging from 90.9 to 94.6 mmHg. The SLBT was performed three times: 15 min after entering the environmental chamber in NOR or HYP, then two times more interspersed by 3-min rest. Peripheral oxygen saturation (SpO_2_) and heart rate (HR) were recorded at four time points: after the initial 15-min rest inside the chamber and immediately after each SLBT. Across the cohort, the balance performance was 7.1% better during NOR than HYP (*P* < 0.01, $\eta_{\text{p}}^{2}$ = 0.58). However, the performance of the elite group was not impaired by HYP, whereas the sub-elite group performed worse in the HYP condition on both legs (DL: *P* = 0.02, *d* = 1.23; NDL: *P* = 0.01, *d* = 1.43). SpO_2_ was lower in HYP than NOR (*P* < 0.001, $\eta_{\text{p}}^{2}$ = 0.99) with a significant decline over time during HYP. HR was higher in HYP than NOR (*P* = 0.04, $\eta_{\text{p}}^{2}$ = 0.25) with a significant increase over time. Acute exposure to normobaric hypoxia detrimentally affected the balance performance in sub-elite but not elite basketball players.

## Introduction

Field- and court-based sports require players to rapidly accelerate, decelerate, and change direction during matches, in order to perform repeated sprints, shuffles, and jumps (Abdelkrim et al., 2010; Pojskic et al., 2018). The resulting physiological and mechanical loading results in fatigue that detrimentally affects postural and motor control, which in turn leads to poorer balance, impaired athletic performance, and increased injury risk (Myklebust et al., 1998; Paillard, 2012; Ruzic et al., 2014). One could, therefore, suggest that balance is a critical determinant of athlete performance in field-based (e.g., football and rugby) and court-based (e.g., basketball and handball) team sports.

In addition to fatigue, several additional factors may negatively influence balance, including sensory alternation, dehydration, and exposure to acute hypoxia (Hoshikawa et al., 2010; Kraemer et al., 2011; Paillard, 2012; Ruzic et al., 2014). It has been well-documented that exposure to moderate and high altitude detrimentally affects various aspects of physical performance in team sports, including repeated sprint ability, running endurance, distance covered, and number of high-intensity sprints during matches (Hamlin et al., 2008; Valenzuela et al., 2019). Moreover, it is well-known that postural control and balance are altered during hypoxic exposure (Degache et al., 2020).

However, the majority of studies exploring the effect of hypoxia on balance have been conducted on aircraft crew members, mountaineers, or healthy active subjects in field-based and laboratory-based settings (Nordahl et al., 1998; Degache et al., 2020). Negative effects of hypobaric hypoxia on balance ability and postural control have been demonstrated in these populations at high altitudes (>3,500 m) (Stadelmann et al., 2015; Bruyneel et al., 2017) and in normobaric hypoxia (Nordahl et al., 1998; Cymerman et al., 2001; Wagner et al., 2011; Drum et al., 2016). Given that high-altitude training has become an increasingly popular training method for team sports (Girard et al., 2013) and that some national, professional, and University teams in ball and court sports (e.g., basketball, baseball, soccer, and American football) may train at moderate (2,300–3,500 m) and high (3,500–5,500 m) altitudes (Kraemer et al., 2011; Gore et al., 2013), improving understanding of the extent to which hypoxic exposure affects balance in team sports is pertinent.

Although it is well-known that balance is more altered in hypobaric than normobaric hypoxia (~1,700–3,000 m above sea level) (Degache et al., 2020), it is also of practical interest to investigate whether exposure to normobaric hypoxia at a simulated altitude of ~3,800 m above sea level would negatively affect balance in well-trained team-sport athletes. In other words, it might reveal a potential adaptation stimulus that could be included in balance training and facilitated by high-altitude chambers. Furthermore, evidence is lacking regarding the influence of player performance level, which has been shown to modulate balance performance both in normoxia (Paillard et al., 2011; Pojskic et al., 2020) and in mountain climbers at high altitudes (3,200 m; Bruyneel et al., 2017).

Moreover, several studies examining single-leg balance performance in normoxia using an omni-axial balancing board have reported good relative and absolute reliability of measurements (e.g., ICC > 0.70 and CV% <10%; Wojtyczek et al., 2014; Hildebrandt et al., 2015; Pojskic et al., 2020). However, there is a lack of studies that have reported reliability data when testing balance in hypoxia using an omni-axial balancing board. Given that an appropriate measurement reliability is important to detect any changes in test performance (Hopkins, 2004; Pojskic et al., 2020), it is important that reliability is also determined in hypoxic conditions.

To the best of our knowledge, no study has investigated the influence of acute hypoxic exposure on balance ability in highly trained athletes active in team sports demanding high levels of dynamic balance and agility. Therefore, the purpose of this study was to investigate whether an acute exposure to normobaric hypoxia would be detrimental to balance performance in elite and sub-elite basketball players. Secondary aims were to investigate whether player performance level modulated balance performance in hypoxia and whether the variability of repeated single-leg balance tests (SLBTs) was greater in hypoxia than normoxia. We expected that an acute exposure to normobaric hypoxia would be less detrimental to balance in the elite than the sub-elite group (Paillard et al., 2011; Bruyneel et al., 2017; Pojskic et al., 2020). Furthermore, it was hypothesized that the balance test would be a valid, reliable, and useful testing tool in assessing balance in basketball players in both conditions (Hildebrandt et al., 2015; Pojskic et al., 2020).

## Methods

### Subjects

The study included 18 highly trained, national-level, male basketball players: nine players who competed at the national senior level (elite group) and nine players (sub-elite group) who competed in U18 national competitions (Table 1). No participants had reported a history of neuromuscular disease or injuries in the previous 6 months, and participants had not undertaken specific balance or high-altitude training in the previous 6 months. Two days prior to the experimental visits, subjects were asked to avoid sleep deprivation, to refrain from high-intensity training, and to avoid tobacco, alcohol, and caffeine. To maintain adequate hydration, players were allowed to drink water *ad libitum* in each experimental condition. The study was approved by the Regional Ethical Review Board (2016-456-31). All participants were informed of the purpose, benefits, and risks of the investigation before providing written informed consent to participate. Parents or guardians provided informed consent for participants under 18 years of age.

**Table 1:** Descriptive characteristics of the elite (*N* = 9) and sub-elite (*N* = 9) basketball players.

**Variables**	**Total**	**Elite**	**Sub-elite**		
	**Mean ± SD**	**Mean ± SD**	**Mean ± SD**	**ES (Cohen's *d*)**	**CI 95% of Cohen's *d***
Age (years)	19.5 ± 2.9	21.8 ± 2.5	17.2 ± 0.4*	2.54	1.25 to 3.80
Playing experience (years)	6.9 ± 2.1	8.7 ± 1.6	5.2 ± 0.4*	2.83	1.47 to 4.16
Body weight (kg)	83.9 ± 9.2	84.5 ± 10.2	83.4 ± 8.6	0.11	−0.81 to 1.03
Body height (m)	1.89 ± 0.08	1.89 ± 0.09	1.89 ± 0.08	0.00	−0.05 to 0.05
Body mass index (kg/m^2^)	23.3 ± 1.5	23.4 ± 1.5	23.2 ± 1.6	0.15	−0.77 to 1.07
Systolic blood pressure (mmHg)	131 ± 10	129 ± 11	132 ± 8	0.32	−0.61 to 1.24
Diastolic blood pressure (mmHg)	78 ± 7	80 ± 7	76 ± 8	0.56	−0.39 to 0.49

*ES, effect size; CI 95%, 95% confidence interval; T1, collected immediately before entering the chamber; T2, collected after exposure inside the chamber; T3, collected after 3-min rest outside the chamber.*

*Significantly different from the high-level group. P < 0.05.

### Study Design

**Figure 1:**
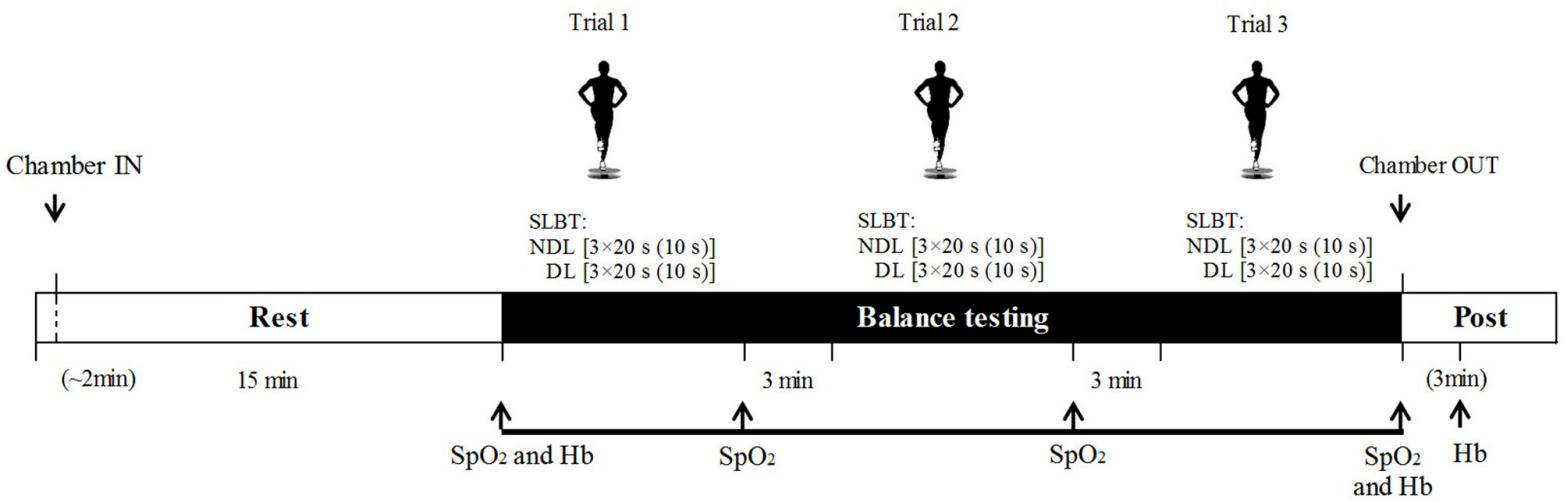
Testing protocol indicating single-leg balance tests, oxygen saturation (SpO_2_), and heart rate (HR) assessment in both conditions: normobaric hypoxia (HYP) and normoxia (NOR). Hemoglobin (Hb) was assessed only in HYP. For balance trials, number of repetitions, repetition duration, and resting periods are represented as follows: leg [repetitions × repetition duration (inter-set recovery)]. DL, dominant leg; NDL, non-dominant leg.

The study was conducted with a randomized, single-blind, cross-over design. To assess the influence of performance level on balance performance in hypoxia, participants were dichotomized into two sub-groups (elite vs. sub-elite). Testing took place over three sessions: a familiarization session and two experimental conditions during which participants performed a test protocol consisting of several attempts of an SLBT on each leg, in a 50 m^3^ altitude chamber (Hypoxico, New York, USA) in normoxia [NOR, ~ sea level] and normobaric hypoxia (HYP: ~3,800 m above sea level, a.s.l, Figure 1). The chamber delivered 1,500 L/min dehumidified air with FiO_2_ 13.0% and PiO_2_ ranging from 90.9 to 94.6 mmHg in HYP and FiO_2_ 20.9% and PiO_2_ ranging from 146.7 to 150.4 mmHg in NOR. In HYP, FiO_2_ was checked at the beginning and end of each trial; a tolerance of 0.3% FiO_2_ was deemed acceptable performance. Ambient pressure was not significantly different between HYP and NOR trials (*P* = 0.21). The temperature controller was set to 20°C for all trials. Good relative reliability (ICC > 0.70) has been established for SLBTs performed on omni-axial balance boards in normoxic conditions (Hildebrandt et al., 2015; Pojskic et al., 2020). Given that this study employed normobaric hypoxia, the reliability of the test equipment would not be expected to differ in the hypoxic condition, although the reliability of execution of the test by participants may be affected, and was therefore investigated as a secondary aim.

Experimental visits were separated by at least 48 h and took place during the competitive season. To avoid anticipatory effects, subjects were blinded to the environmental conditions in the chamber (i.e., all digital panels were concealed from view) and the purpose of measuring various physiological responses. We chose the simulated high altitude to be over 3,000 m a.s.l, as it is the altitude threshold at which reductions in SpO_2_ start to detrimentally affect postural control and perceptual-motor performance (Fowler et al., 1987). This was to ensure that the hypoxic stimulus would be sufficient to affect balance performance in basketball players, presumed to have well-developed balance abilities (Curtolo et al., 2017).

### Testing Protocol

On the familiarization day, body weight, height, blood pressure, and resting heart rate (HR) of subjects were measured (Table 1). After that, the subjects were familiarized to the SLBT. On the second and third sessions, subjects performed a standardized balance test protocol 15 min after entering the environmental chamber either in the NOR or HYP condition. The test protocol consisted of three trials interspersed by 3-min rest. Each trial comprised three 20 s attempts of the SLBT with 10 s recovery, performed first on the dominant (DL) followed by the non-dominant (NDL) leg (Figure 1). Special attention was paid to ensure an accurate foot placement on the balance platform and maintain a stable body posture and hand positioning. The participants were instructed to refrain from any other breathing methods such as hyperventilation.

### Single-Leg Balance Test

The SLBT employed a single-leg upright stance on an omni-axial balance board and was chosen to provide unstable, dynamic conditions to test balance ability requiring complex rearrangement of postural control (Valle et al., 2015; Petró et al., 2017; Pojskic et al., 2020). We assumed that this complexity would ensure discriminative power under different conditions (i.e., NOR vs. HYP) in balance-proficient athletes playing at different levels. The SLBT was executed using an MFT challenge disk system (TST Trendsport, Grosshöflein, Austria), which assesses the ability of an individual to maintain stability on a multiaxial tilting platform and produces a “balance index” ranging from 1 to 5, where a lower index represents less deviation from the horizontal plane and thus better balance. The procedure for the SLBT has been reported in detail elsewhere (Pojskic et al., 2020). Briefly, the DL of subjects was determined based on which leg a subject uses to kick a ball (van Melick et al., 2017). Then, without shoes, subjects stood on one leg in the middle of the balance plate with a slightly flexed knee, kept their hands on their waist, and were instructed to keep the platform in a horizontal position. The SLBT was performed for 20 s, but restarted if subjects made two “mistakes” (such as touch the floor or balance plate with the free foot). In addition, if only one mistake was made within a 20-s attempt, then the performance was penalized by adding 0.2 of the balance index to the produced result. The mean scores of the three attempts for each of the three trials were used to calculate test–retest reliability coefficients. To analyze the differences between experimental conditions, the mean score of the three trials was used.

### Pulse Oximetry, Heart Rate, and Hemoglobin Monitoring

During the two experimental conditions, SpO_2_ and HR were assessed using wireless pulse oximeters (Wristox 3100; Nonin, Plymouth, MN, USA) placed on the middle finger and recorded manually at four time points: after the initial 15 min rest inside the chamber and then immediately after each SLBT trial. The display on the oximeters was covered to assist with blinding. Hb samples were collected only in HYP at three time points: (a) immediately before entering the chamber; (b) after exposure inside the chamber; and (c) after 3 min rest outside the chamber. Capillary blood was sampled using a 21G safety lancet (Sarstedt, Nuembrecht, Germany) collected into microcuvettes, and Hb was analyzed in triplicate using a Hb 201 Analyzer (H31216, Hemocue, Ängelholm, Sweden).

### Statistical Analyses

The sample size was estimated *a priori* using previously published SLBT index means and standard deviations (SDs; Hildebrandt et al., 2015; Pojskic et al., 2020). Using G-Power software (version 3.1.9.2; Heinrich Heine University Dusseldorf, Dusseldorf, Germany), we estimated that nine subjects (df = 8) would provide an appropriate sample size for paired-samples differences (*P* ≤ 0.05, power = 0.90).

Data are presented as means and SDs. Normality was assessed using the Shapiro-Wilk test. A four-factor mixed ANOVA with three within-subject factors [condition (NOR vs. HYP), leg used (DL vs. NDL), and SLBT trials (1–3)] and one between-subject factor (elite vs. sub-elite) was used to analyze effects on balance. A three-factor ANOVA was used to evaluate the effect of group, condition, and trial on SpO_2_ and HR. Bonferroni *post-hoc* tests were used for pairwise comparisons. Partial eta squared ($\eta_{\text{p}}^{2}$) was calculated for the ANOVA main effects, with effect sizes interpreted as follows: >0.02 (small), >0.13 (medium), and >0.26 (large). To investigate differences in balance between playing levels and between NOR and HYP, *t*-tests for independent and dependent samples were used. Cohen's *d* effect sizes were also calculated and interpreted as follows: <0.2 (trivial), 0.2–0.6 (small), 0.6–1.2 (moderate), 1.2–2.0 (large), and >2.0 (very large) differences (Cohen, 1988; Hopkins, 2000). Analysis of relative reliability was performed for all balance tests by calculating the intraclass correlation coefficient (ICC, model 3.1). The absolute reliability (within-subject variation) was established using the coefficient of variation (CV%) expressed in percentage (Hopkins, 2000). Usefulness was computed by comparing typical error (TE) and the smallest worthwhile change (SWC) of the balance index (Hopkins, 2000). SWC was derived from between-subject SD multiplied by 0.2 (SWC_0.2_; small effect) or 0.5 (SWC_0.5_; moderate effect) (Cohen, 1988). A TE below SWC indicates test usefulness as “good,” and TE similar to SWC is rated “acceptable.” If TE is higher than SWC, it is deemed to have “marginal” usefulness (Hopkins, 2004). Statistical significance for all tests was set at *P* ≤ 0.05. Statistical analyses were performed using SPSS®24.0 (IBM, New York, USA).

## Results

### Reliability of Balance Tests

The relative reliability for the SLBT was high in each condition (DL and NDL, NOR and HYP), with ICC ranging from 0.73 to 0.90. The absolute reliability expressed as CV% for the SLBTs was 6.95% and 7.58% in NOR and 8.99% and 6.59% in HYP condition, for DL and NDL, respectively. In both the NOR and HYP conditions, the TE exceeded SWC_(0.2)_, whereas it was below the SWC_(0.5)_ for all balance tests (see Supplementary Table 1). No significant differences in performance between legs or within trials were detected in any condition for any group (Table 2).

**Table 2:** Balance index scores for the high and low playing level groups in NOR and HYP condition.

**Condition**	**Total**	**Elite**	**Sub-elite**	**Between-subjects differences**		**Within-subjects differences**	
						**Elite**	**Sub-elite**
	**Mean ± SD**	**Mean ± SD**	**Mean ± SD**	**ES (Cohen's *d*)**	**CI 95% of Cohen's *d***	**ES (Cohen's *d*)**	**ES (Cohen's *d*)**
NOR-DL	3.34 ± 0.36	3.22 ± 0.39	3.47 ± 0.30^†^	0.71	−0.25 to 1.66	0.71	1.65
HYP-DL	3.65 ± 0.38	3.45 ± 0.35	3.86 ± 0.31*	1.23	0.20 to 2.23		
NOR-NDL	3.45 ± 0.51	3.29 ± 0.46	3.61 ± 0.52^†^	0.64	−0.31 to 1.58	0.12	0.80
HYP-NDL	3.59 ± 0.48	3.31 ± 0.47	3.88 ± 0.29*	1.44	0.37 to 2.47		

*SLBT, single-leg balance test; ES, effect size; CI 95%, 95% confidence interval. Mean ± SD (range) of balance index (performance) across the three trials in both normoxia (NOR) and normobaric hypoxia (HYP) for the dominant (DL) and non-dominant (NDL) legs.*

*Significantly different from the elite group.

*†Significantly different from HYP condition. P <0.05*.

### Effects of Hypoxia on Balance Performance

**Figure 2:**
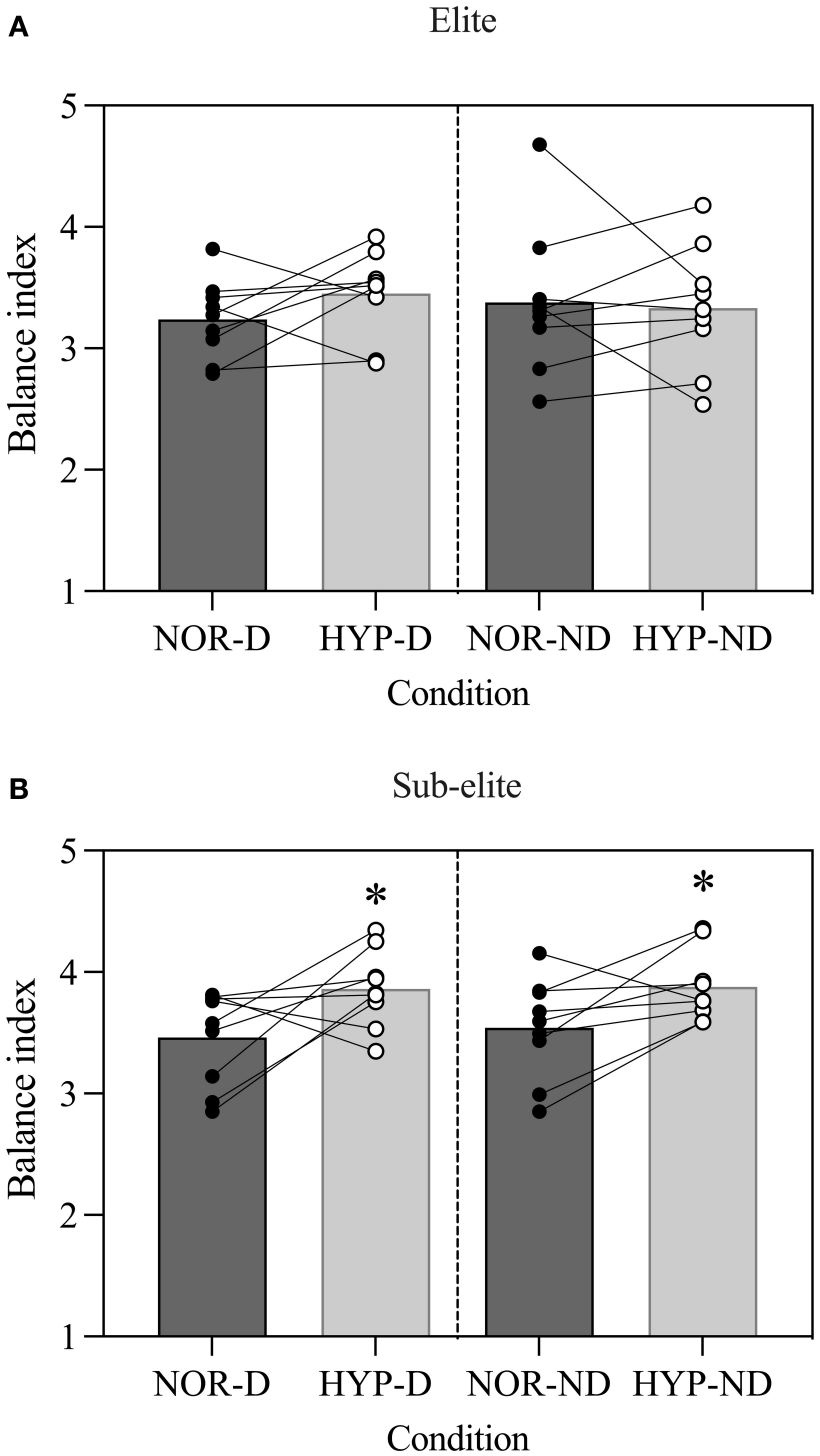
Acute effects of normobaric hypoxia on a single leg balance test performance in **(A)** elite and **(B)** sub-elite basketball players. NOR, normoxic condition; HYP, normobaric hypoxic condition; D, dominant leg; ND, non-dominant leg. *Values significantly different from those obtained in NOR; *P* < 0.05.

Balance performance was 7.1% better during NOR (3.40 ± 0.43) than HYP (3.62 ± 0.44) (*F* = 22.3, *P* = 0.00, $\eta_{\text{p}}^{2}$ = 0.58; Figure 2). The balance performance was also 10.4% better in the elite (3.32 ± 0.41) than the sub-elite group (3.70 ± 0.37) (*F* = 4.85, *P* = 0.04, $\eta_{\text{p}}^{2}$ = 0.23; Figure 2). Further exploration revealed that only the elite group performed better than the sub-elite group in the HYP condition, on both DL [*t*_(16)_ = 2.62, *P* = 0.019, *d* = 1.23] and NDL [*t*_(16)_ = 2.78, *P* = 0.01, *d* = 1.44] (Table 2).

In the elite group, no differences in balance were detected between NOR and HYP, for DL [*t*_(8)_ = 2.15, *P* = 0.06, *d* = 0.71] or NDL [*t*_(8)_ = 0.37, *P* = 0.72, *d* = 0.12]. Conversely, the sub-elite group had poorer balance on DL [*t*_(8)_ = 4.96, *P* < 0.00, *d* = 1.65] and NDL [*t*_(8)_ = 2.40, *P* = 0.04, *d* = 0.80] in HYP than NOR (Table 2).

### Pulse Oximetry, Heart Rate, and Hemoglobin Concentration

The SpO_2_ was lower in HYP than NOR (*F* = 14.9, *P* < 0.00, $\eta_{\text{p}}^{2}$ = 0.99; Supplementary Figure 1A) with a mean difference of ~15% [95% CI (14.0–15.7)]. However, the group × condition interaction revealed lower SpO_2_ in elite compared with sub-elite players in HYP (*F* = 7.26, *P* = 0.02, $\eta_{\text{p}}^{2}$ = 0.31). Regardless of condition and group affiliation, SpO_2_ decreased across the sampling time points (*F* = 3.98, *P* = 0.01, $\eta_{\text{p}}^{2}$ = 0.20, Supplementary Figure 1A), but without significant pairwise differences between them. The HR response was higher in HYP than NOR (*F* = 4.85, *P* = 0.04, $\eta_{\text{p}}^{2}$ = 0.25, Supplementary Figure 1B) with a mean difference of ~6 beats/min [95% CI (0.2–11.4)]. Significant differences in HR were found 15 min after entering the chamber compared with the first, second, and third trials in both conditions (Supplementary Figure 1B). There were no differences in HR response between the groups (*P* = 0.64). No significant changes were found in Hb concentration between sampling time points in HYP (pre: 146 ± 9 g × L^−1^, after: 148 ± 9 g × L^−1^, and 3 min post-recovery: 147 ± 10 g × L^−1^; *F* = 1.45, *P* = 0.26, $\eta_{\text{p}}^{2}$ = 0.16). No significant interaction between the group and condition was found for Hb (*F* = 1.98, *P* = 0.17, $\eta_{\text{p}}^{2}$ = 0.21).

## Discussion

This is the first study that has investigated the effects of acute exposure to high altitude on single-leg balance performance in highly trained basketball players. The main findings of this study are that: (a) acute exposure to normobaric hypoxia equivalent to ~3,800 m a.s.l. impaired balance performance in sub-elite but not elite basketball players and (b) the SLBT showed good reliability in normobaric hypoxia that was comparable with the reliability in normoxia.

### Effects of the Exposure on Balance Performance

In this study, normobaric hypoxia detrimentally affected the balance (~7%), when subjects were exposed to a simulated altitude of ~3,800 m. This is in line with previous studies that have demonstrated elements of impaired balance, such as postural stability (Cymerman et al., 2001; Degache et al., 2020) and body sway (Nordahl et al., 1998; Wagner et al., 2011) in simulated altitude (2,400 m to 5,500 m) vs. normoxia, with exposure durations of a few minutes to 24 h in non-athletic populations. However, this study corroborates and extends these findings by revealing detrimental effects of HYP on balance even in highly trained basketball players who engage in activities demanding high levels of balance. It is well-known that hypoxia can negatively affect the sensory (e.g., the vestibular, proprioceptive, and visual) and central nervous systems, which are eminently sensitive to decrements in tissue oxygenation (Pickard and Gradwell, 2008) leading to impaired neuromuscular coordination, postural stability, and balance (Degache et al., 2020).

Interestingly, this study showed that the detrimental effect of HYP on balance was modulated by the playing level of subjects, with those playing at a higher level having better balance in hypoxia. Previous studies conducted in other sports (e.g., curling, football, and surfing) in normoxia showed advanced balance in athletes competing at higher playing levels, suggesting that balance may be sensitive to training status, playing experience, and/or sport- and movement-specific adaptations (Paillard et al., 2011; Pojskic et al., 2020). In this study, given that there was no difference in balance between HYP and NOR in the elite group, we could assume that both longer and advanced basketball training and competition might have induced advanced adaptation of the neuromuscular system, which in turn was less susceptible to the adverse effects of the HYP condition (Bruyneel et al., 2017). Although there was a significant age difference between the elite (21.8 ± 2.5 years) and sub-elite (17.2 ± 0.4 years) groups, we cannot consider a difference in the maturation of the sensory systems as a crucial factor contributing to advanced balance performance in the higher level group. We reason this based on two arguments: firstly, because there was no difference between groups in SLBT performance in NOR condition, and secondly, because the visual and vestibular afferent systems, that are responsible for providing accurate sensory inputs and maintenance of postural control, reach adult level at 15–16 years of age (Hirabayashi and Iwasaki, 1995; Steindl et al., 2006).

We can speculate that the elite group may have been better able to compensate for the additional demands on balance performance in hypoxia, potentially by reducing spinal reflex activity and the excitability of the spinal α-motoneurons, that are increased by reduced oxygen delivery to target tissues (Lundby et al., 2009). As a result, reduced activation of the muscles encompassing the joints (e.g., the ankles and knees) may inherently prevent unwanted and uncontrollable joint oscillations when performing the SLBT (Keller et al., 2012). Additionally, considering that vision is one of the first senses to be affected by acute hypoxia (Nordahl et al., 1998; Degache et al., 2020) and that the SLBT was performed with open eyes, the elite group may have been able to compensate for reduced vision capacity through greater contributions from vestibular and proprioceptive sensory information (Paillard et al., 2002, 2011; Pojskic et al., 2020). In brief, it is well-documented that the role of the visual system on balance performance decreases with higher playing level in athletes (Paillard and Noé, 2006; Paillard et al., 2006). Given that successful basketball game play requires players to control the ball and move down the court without watching it while observing the situation in the game (e.g., movement of teammates and opponent), it means that elite players, as a result of long-term adaptation to basketball training, became less dependent on vision and more on their proprioception when maintaining balance (Paillard, 2019).

In contrast, less experienced athletes rely more on vision for maintaining balance (Paillard et al., 2002, 2011; Paillard and Noé, 2006), and consequently, their balance may have been more negatively affected by reduced SpO_2_ (Bruyneel et al., 2017). This can be supported by the fact that sub-elite players experienced a bigger deterioration in balance performance despite displaying milder reductions in SpO_2_ than their elite counterparts. Moreover, sub-elite players had higher HR across all trials which might indicate a higher cardio-ventilatory response and subsequent negative effects on postural control. It is known that higher hyperventilation increases the frequency of respiratory movements which in turn increases postural sway and decreases balance performance (Hodges et al., 2002; Malakhov et al., 2014). Collectively, we can assume that elite players had advanced adaptation that enabled them to efficiently integrate inputs from the visual, vestibular, and somatosensory systems required for an adequate motor response to maintain balance and postural control (Wagner et al., 2011).

### SLBT Validity, Reliability, and Usefulness

In this study, the SLBT showed good relative reliability both in normoxia and normobaric hypoxia. These results were comparable with the reliability in previous studies that have used an omni-axial balancing board and single-leg dynamic balance measurements in healthy and sporting populations (ICC range 0.76–0.97; Hildebrandt et al., 2015; Pojskic et al., 2020). To establish stable measurement conditions in hypoxia as a prerequisite for high measurement reliability, subjects performed the SLBT after 15' acclimation in the chamber. Moreover, good relative reliability could be also explained by high between-subjects variability in SLBT with those with higher playing level and longer playing experience having better balance than their sub-elite counterparts (Table 2; Pojskic et al., 2020). Furthermore, the absolute reliability (e.g., within-subjects variability) obtained in both HYP and NOR was in line with previous data for similar tests (CV% = 7–8%; Pojskic et al., 2020). The “acceptable” values could be attributed to players having the required test-dependent motor proficiency that is developed in agility-based team sports (Curtolo et al., 2017). In addition, it seemed that using the omni-axial balancing board as a simple testing tool for testing balance in an upright stance, which is a natural position for basketball players, could reduce potential covariates of performance (i.e., measurement error) and consequently enable better test–retest reliability and thus the potential to attribute results to the condition (NOR vs. HYP), instead to the testing equipment or protocol (Sekulic et al., 2017; Pojskic et al., 2020). Given that the SLBT did not show significant systematic change over the three trials, it means in practice that as long as the familiarization is conducted as described, two SLBT trials would be sufficient to obtain reliability data (Pojskic et al., 2020).

Moreover, the usefulness of the tests was estimated by a trial-to-trial change in balance performance and by comparing the TE and both the SWC_(0.2)_ and SWC_(0.5)_. For all tests, SWC_(0.2)_ was shown to be “marginal” (i.e., TE > SWC) in both the NOR and HYP conditions. In contrast, in both conditions, SWC_(0.5)_ exceeded TE, showing “good” usefulness. In summary, the SLBT can be utilized to detect moderate changes that exceed 0.5 × SD, in both the NOR and HYP conditions (Hopkins, 2004). Finally, SLBT performed only in HYP could be considered to have greater power to discriminate balance performance between playing levels. This is not totally unexpected, because the power of a test to discriminate playing levels is higher if the test protocol and conditions are sport specific and more demanding (Pojskic et al., 2020). The results are in agreement with Bruyneel et al. (2017) who recently reported differences in postural stability between two expertise groups of mountaineers at 3,200 m, but not at 1,500 m, tested immediately after exiting the cable car.

### Limitations

This study has several limitations that must be acknowledged. First, the cross-sectional design limits the extent to which the group differences can be attributed only to the experimental conditions. Second, even though the subjects were blinded to the conditions being exposed to, using a double-blind design could make testing results less likely to be biased by the experimenter. Third, the technological design of the balance plate did not provide information on balance performance in specific mediolateral or anteroposterior directions, only a composite multidirectional balance index. Moreover, the balance plate used does not measure balance performance in basketball-specific movement patterns. Also, the study did not incorporate hypobaric hypoxia, which is most ecologically relevant for high altitude training and competition venues. This study did not evaluate physiological responses related to balance, such as the spinal reflex, brain activity, and muscles activity, which could help to identify mechanisms responsible for the observed differences between the conditions. In addition, even including highly trained players and calculating the minimum number of included subjects before, the sample size was low which limits the potential of the study to generalize the findings to the basketball population. Moreover, the age difference between groups was significant which might affect the results. Finally, the present results are specific to the basketball players chosen for the experiment, so caution should be exercised in generalizing the effects across other groups of athletes competing at high altitudes.

## Conclusion

This study was the first to demonstrate that acute hypoxic exposure detrimentally affects balance performance in well-trained athletes active in team sports demanding high levels of dynamic balance and agility. Furthermore, acute effects of normobaric hypoxia on balance performance were modulated by the playing level with elite players having better resistance to its adverse effects than their sub-elite counterparts. The SLBT showed good reliability and usefulness in both normoxic and normobaric hypoxic conditions. However, the SLBT showed sufficient sensitivity to discriminate playing levels only in normobaric hypoxia equivalent to high altitudes (~3,800 m).

Basketball coaches and practitioners should be aware that acute exposure to hypoxia equivalent to high altitudes (~3,800 m) might negatively affect balance in sub-elite players and consequently execution of all activities that require it, such as acceleration and deceleration movements and sport-specific motor tasks (e.g., shooting and passing accuracy, and dribbling speed). Moreover, as acute normobaric hypoxia impairs balance performance, future studies can be designed to explore whether hypoxic balance training has the potential to facilitate faster and more advantageous training adaptation and provide a positive transfer to balance performance in hypobaric hypoxia (i.e., high altitude).

## Data Availability Statement

The raw data supporting the conclusions of this article will be made available by the corresponding author, without undue reservation, to any qualified researcher.

## Ethics Statement

The studies involving human participants were reviewed and approved by the Regional Ethical Review Board in Umeå (2016/456-31). Written informed consent to participate in this study was provided by the participants. Legal guardian/next of kin provided informed consent for participants under 18 years of age.

## Author Contributions

HP contributed to the research concept and study design, literature review, data analysis and interpretation, statistical analyses, manuscript writing, and manuscript editing. HH contributed to the data analysis and interpretation, manuscript writing, and manuscript reviewing/editing. LR-Z contributed to the literature review, data collection, data analysis and interpretation, and manuscript writing. T-HT contributed to the data collection, manuscript writing, and manuscript reviewing/editing. All authors contributed to the article and approved the submitted version.

## Funding

This study was supported by the Swedish Winter Sports Research Centre at Mid Sweden University, which had no role in the design or conduct of this research and by Linnaeus University that financed the open access publication fee.

## Conflict of Interest

The authors declare that the research was conducted in the absence of any commercial or financial relationships that could be construed as a potential conflict of interest.

## Publisher's Note

All claims expressed in this article are solely those of the authors and do not necessarily represent those of their affiliated organizations, or those of the publisher, the editors and the reviewers. Any product that may be evaluated in this article, or claim that may be made by its manufacturer, is not guaranteed or endorsed by the publisher.

## Supplementary Material

Supplementary Figure 1 Acute effects of normobaric hypoxia on oxygen saturation (SpO_2_) and heart rate response (HR). T_1−3_, trials 1–3 of the single-leg balance tests; NOR, normoxic condition; HYP, normobaric hypoxia condition. ^*^Indicates significant difference between elite and sub-elite players, *P* < 0.05. Indicates significant difference from values obtained at first measurement (after the initial 15 min of resting inside the chamber), *P* < 0.05. ^#^Indicates a significant difference between HYP and NOR, *P* < 0.05. ^###^*P* < 0.001.

Supplementary Table 1 Reliability parameters for the SLBT at DL and NDL in NOR and HYP.
