# Supplementary figures and images for: Acute Exposure to Normobaric Hypoxia Impairs Balance Performance in Sub-elite but Not Elite Basketball Players
Source: Front Physiol. 2021 Oct 27;12:748153. doi: 10.3389/fphys.2021.748153 (PMC8578732; doi:10.3389/fphys.2021.748153)

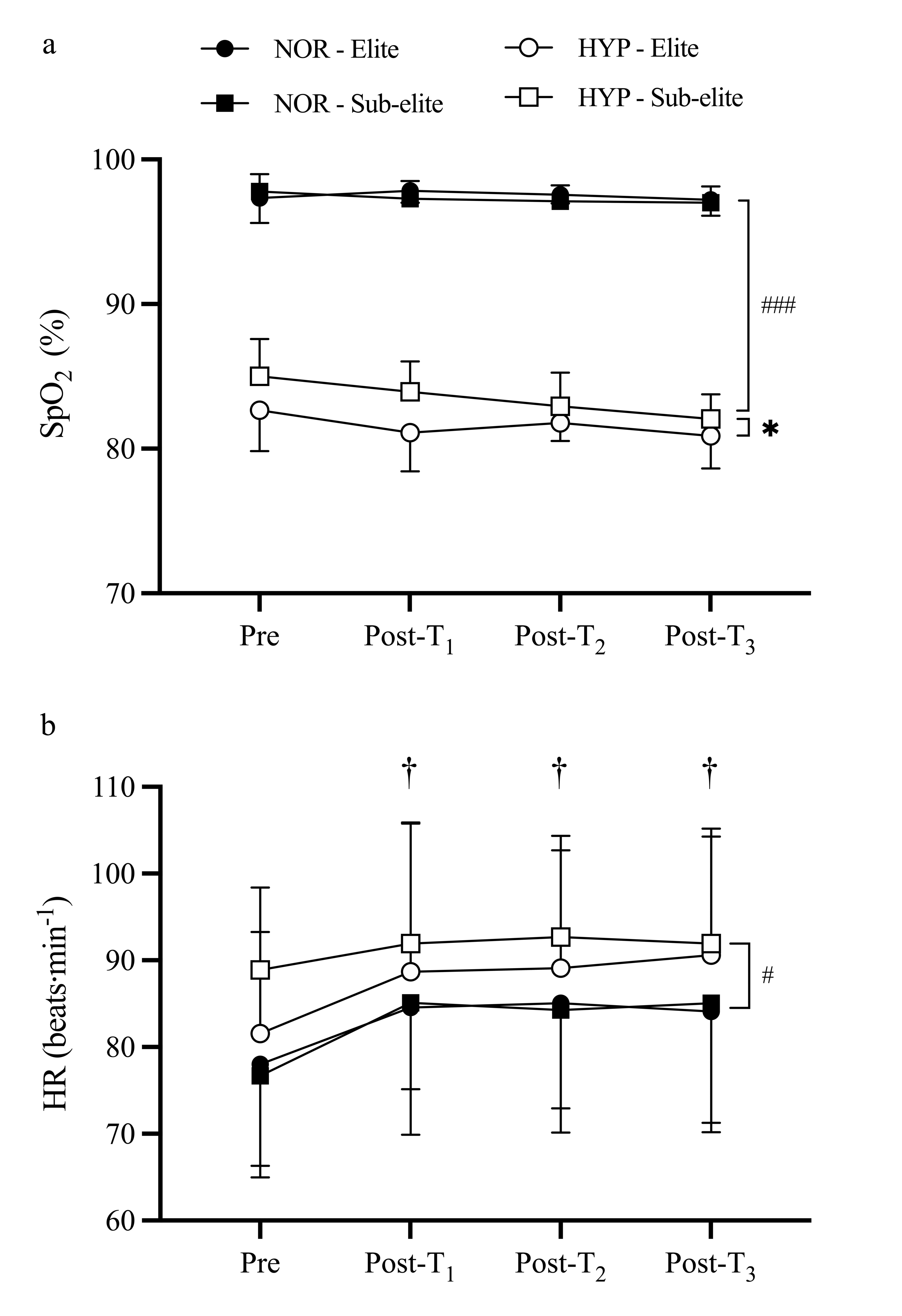

Supplement: Supplementary Figure 1 — Acute effects of normobaric hypoxia on oxygen saturation (SpO2) and heart rate response (HR). T1−3, trials 1–3 of the single-leg balance tests; NOR, normoxic condition; HYP, normobaric hypoxia condition. *Indicates significant difference between elite and sub-elite players, P < 0.05. Indicates significant difference from values obtained at first measurement (after the initial 15 min of resting inside the chamber), P < 0.05. #Indicates a significant difference between HYP and NOR, P < 0.05. ###P < 0.001. [file Image_1.JPEG]
