# Supplementary material for: Acute Exposure to Normobaric Hypoxia Impairs Balance Performance in Sub-elite but Not Elite Basketball Players
Source: Front Physiol. 2021 Oct 27;12:748153. doi: 10.3389/fphys.2021.748153 (PMC8578732; doi:10.3389/fphys.2021.748153)
Supplement: Supplementary Table 1 — Reliability parameters for the SLBT at DL and NDL in NOR and HYP. [file Table_1.doc]

**Supplementary table 1. Reliability parameters for the SLBT at DL and NDL in NOR and HYP.**

|  | Balance index | | |  |  |  |  |  |  |
| --- | --- | --- | --- | --- | --- | --- | --- | --- | --- |
| **Condition** | **Trial 1**  mean±SD (range) | **Trial 2**  mean±SD (range) | **Trial 3**  mean±SD (range) | **CV%**  (ws) | **CV%**  (bs) | **ICC**  **(95%CI)** | **TE** | **SWC_(0.2)_** | **SWC_(0.5)_** |
|  |  |  |  |  |  |  | balance index | | |
| NOR-DL | 3.41 ± .38 (2.83-4.01) | 3.37 ± .41 (2.67-4.07) | 3.26 ± .45 (2.24-4.13) | 6.95 | 10.58 | 0.84 (0.64 - 0.93) | 0.11 | 0.07 | 0.18 |
| HYP-DL | 3.74 ± .43 (2.93-4.48) | 3.73 ± .48 (2.82-4.46) | 3.49 ± .52 (2.61-4.50) | 8.99 | 10.59 | 0.73 (0.40 - 0.89) | 0.18 | 0.10 | 0.23 |
| NOR-NDL | 3.54 ± .56 (2.73-4.76) | 3.42 ± .53 (2.55-4.56) | 3.41 ± .58 (2.36-4.72) | 7.58 | 14.71 | 0.88 (0.75 - 0.95) | 0.17 | 0.07 | 0.18 |
| HYP-NDL | 3.70 ± .49 (2.53-4.23) | 3.51 ± .49 (2.70-4.49) | 3.56 ± .57 (2.33-4.56) | 6.59 | 13.36 | 0.90 (0.78 - 0.96) | 0.16 | 0.09 | 0.21 |

Legend: SLBT = single-leg balance test;Mean ± SD (range) ofbalance index (performance) acrossthethreetrials in bothnormoxia (NOR)andnormobarichypoxia (HYP) for the dominant (DL) andnon-dominant (NDL)legs.CV%(ws) = within-subjects coefficientofvariation; CV%(bs) = between-subjects coefficientofvariation; ICC = Intra-class coefficient, 95%CI=95% confidence interval; TE = typical error of the measurement; SWC_0.2_ = smallest worthwhile change (0.2 x SD); SWC_0.5_ = smallest worthwhile change (0.5 x SD).
